# EVALUATING PSYCHOMETRIC PROPERTIES OF THE SOCIAL FRAIL INDEX IN COMMUNITY-DWELLING OLDER ADULTS

**DOI:** 10.1093/geroni/igae098.2606

**Published:** 2024-12-31

**Authors:** Inthira Roopsawang, Suparb Aree-Ue, Teepatad Chintapanyakun

**Affiliations:** Mahidol University, Bangkok, Krung Thep, Thailand; Mahidol University, Bangkok, Krung Thep, Thailand; Mahidol University, Bangkok, Krung Thep, Thailand

## Abstract

Social frailty has become a concern issue in older adults since it has a negative impact on older adults’ health with various dimensions including physical and mental health leading to a decline in the ability to self-care, increasing depression, or engaging in social activities. The original version of the Social Frail Index—Japanese— has been translated into various languages, but no information is available in Thai language. The aim of this cross-sectional study, therefore, was to evaluate the valid and psychometric properties of the Social Frail Index Thai version to further explore social frailty and its associated health consequences in Thai older adults. After translation using back translation technique, the Social Frail Index was confirmed for its validity and reliability in 380 dwelling-community Thai older adults living in four regions in Thailand. Participants responded to the Demographic and Health Information Questionnaire and the Social Frail Index. Results revealed that the item-level content validity index (I-CVI) and scale-level content validity index based on the universal agreement method (S-CVI/UA) were 0.96 and 0.80, respectively. The construct validity— Confirmatory Factor Analysis—confirmed that the model showed a well fit for the whole model. The factor loading ranged from.331 to.969 with a variance of 11.00-93.90. For its reliability, inter-ratter reliability of Kappa coefficient was.87 and internal consistency reliability of Cronbach Alpha was.80, indicating a good level. It can be concluded that the Social Frail Index Thai version was valid and reliable for evaluating social frailty in Thai older adults.

